# SOX4 contributes to the progression of cervical cancer and the resistance to the chemotherapeutic drug through ABCG2

**DOI:** 10.1038/cddis.2015.290

**Published:** 2015-11-19

**Authors:** R Sun, B Jiang, H Qi, X Zhang, J Yang, J Duan, Y Li, G Li

**Affiliations:** 1 Cancer Research Institute, Central South University, Changsha, China; 2 Key Laboratory of Carcinogenesis, National Health and Family Planning Commission, Changsha, China; 3 Key Laboratory of Carcinogenesis and Cancer Invasion, Ministry of Education, Changsha, China; 4 Collaborative Innovation Center of Molecular Diagnosis and Laboratory Medicine in Henan Province, School of Laboratory Medicine, Xinxiang Medical University, Xinxiang, China; 5 The Third Affiliated Hospital, Xinxiang Medical University, Xinxiang, China

## Abstract

SOX4, a member of the SOX (sex-determining region Y-related HMG box) transcription factor family, has been reported to be abnormally expressed in a wide variety of cancers, and to exert a pleiotropic function. However, its function in progression of cervical cancer (CC) remains unknown. In this study, we found that SOX4 was highly expressed in CC cells and tissues, and overexpression of SOX4 in CC CaSki cells enhanced tumor clone formation and cell proliferation, and accelerated cell cycle progress. Meanwhile, downregulation of SOX4 by shRNA in CaSki cells inhibited cell proliferation, and slowed cell cycle progress, indicating that SOX4 contributes to the development of CC. In addition, SOX4 overexpression by gene transfer reduced the sensitivity of CaSki cells in response to the chemotherapeutic drug cisplatin, and SOX4 downregulation by RNA interference increased the sensitivity of CaSki cells in response to cisplatin. Moreover, SOX4 overexpression upregulated multiple drug resistant gene ABCG2, and SOX4 downregulation inhibited ABCG2 expression. Taken together, these results suggested that SOX4 functions to modulate cancer proliferation by regulation of cell cycle, and inhibit cancer cell sensitivity to therapeutic drug via upregulation of ABCG2. Thus, SOX4 may be a target for CC chemotherapy.

Cervical cancer (CC) is the second most commonly diagnosed cancer and third leading cause of cancer death among females in developing countries. There were an estimated 527 600 new cervical cancer cases and 265 700 deaths worldwide in 2012.^1^ With the change of life behaviors,^2^ younger women are most affected in several countries.^3, 4^ Although the screening techniques and HPV vaccines can reduce the morbidity of CC effectively, the increasing morbidity of CC among young women and the refractoriness of terminal CC appears to be new problems.

The SOX (sex-determining region Y (SRY)-related high-mobility-group (HMB) box transcription factor) gene family is found throughout the animal kingdom. In vertebrate, at least 20 members of this family have so far been identified.^5^ SOX4 is a 47-kDa protein member of this family encoded by a single exon gene,^6^ and it preferentially binds the ^A^/_T_^A^/_T_CAAAG sequence motif through HMB domain and regulates transcription of target genes.^7, 8^ Similar to many other members of the family, SOX4 has also been identified as a necessary factor in embryonic development. Recently, multiple studies have revealed aberrant expression of SOX4 in several human cancers. It has been reported that SOX4 protein upregulated and exerted an oncogenic function in cancers of prostate cancer,^9^ endometrial cancer^10^ and breast cancer.^11^ In breast cancer, high expression of SOX4 induces epithelial–mesenchymal transition and results in a cancer stem cell-like phenotype in breast cancer cells.^11^ However, a reduced expression of SOX4 was detected in cancer of cutaneous melanoma,^12^ primary gallbladder carcinoma^13^ and colon cancer,^14^ and SOX4 has a cancer suppressor gene role. In cutaneous melanoma, SOX4 expression is low. SOX4 suppresses melanoma cell migration and invasion ability through inhibition of NF-*κ*B p50 expression, and has positively correlated with better patient survival.^12^ Although pleiotropic function of SOX4 is reported, its role in CC is unknown, and this study aimed to fully explore the function and its mechanism of SOX4 in CC.

The resistance to chemotherapeutic drugs remains one of the primary obstacles in cancer treatment. The ability of cells to acquire resistance to multiple drugs is often mediated by overexpression of ATP-binding cassette (ABC) transporters that remove drugs out of the cells and reduce intracellular drug levels.^15^ The human ABCG2 gene is located on chromosome 4 and extends over 68 kb containing 16 exons and 15 introns. The translational start site is found in the second exon and the first exon contains the majority of the 5′ UTR.^16^ ABCG2 proteins are thought to exist on the plasma membrane where they effluxe xenobiotics, such as mitoxantrone, doxorubicin, certain camptothecins and methotrexate.^17, 18, 19, 20, 21^ There are some reports that ABCG2 located on the membranes of mitochondria,^22^ in perinuclear region^23^ or in nuclear as a transcription regulator to modulate the metastasis in lung cancer.^24^ In addition, ABCG2 is also used as a marker for cancer stem cells or cancer-initiating cells in various human cancers.^25, 26^

## Results

### The expression of SOX4 in human cervix epithelium cells and samples

**Figure 1:**
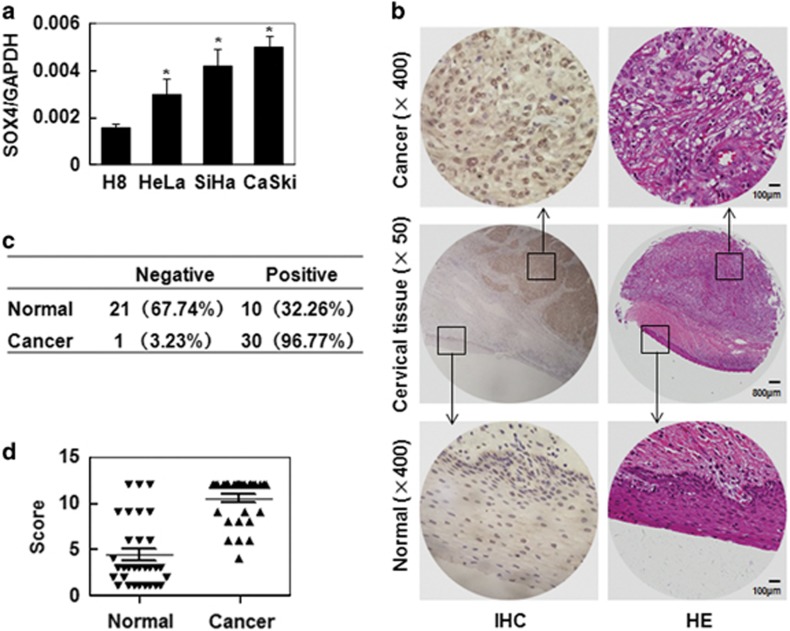
SOX4 expression in cervix cancer cells and tissues. (**a**) qRT-PCR analysis of SOX4 mRNA levels in one normal cervical cell line (H8) and three CC cell lines (HeLa, SiHa and CaSki). (**b**) Representative HE straining and immunohistochemistry (IHC) analysis of SOX4 expression in normal cervical tissue and CC tissue. (**c**) SOX4 staining in 31 cervical squamous cancer specimens and their paired normal cervical epithelial tissues adjacent to cancer. (**d**) A comparison of the IHC scores of SOX4 staining in normal cervix and cervical squamous cancer. SOX4 relative expression levels of each specimen were shown as the means±S.E. in NC and ICC groups. *P<0.05 compared with the normal tissue group

Although SOX4 expression has been discovered in various carcinomas, its role in CC is not well defined. In the present study, we first detected the expression of SOX4 by qRT-PCR in one human normal cervical epithelial cell H8 and three human CC cell lines (HeLa, SiHa and Caski). The results show that SOX4 mRNA was detected in all four cervical epithelia cell lines and increased significantly in the three CC cell lines compared with that in normal cervical epithelial cells (Figure 1a). The expression of SOX4 was analyzed by immunohistochemistry in 31 cervical squamous carcinoma samples and their paired adjacent normal tissues (Figure 1b). SOX4 positive staining localized in nucleus and/or cytoplasm was found in 32.26% (10 of 31) of the normal cervix samples and in 96.77% (30 of 31) of the CC samples (Figure 1c). The average scores of IHC for SOX4 were 4.36±0.65 in normal cervix samples and 10.48±0.43 in CC (Figure 1d). These data indicated that SOX4 was upregulated in CC and it may be involved in the progression of CC.

### SOX4 promoted the tumor formation and the proliferation of cervical cancer cells *in vivo*

**Figure 2:**
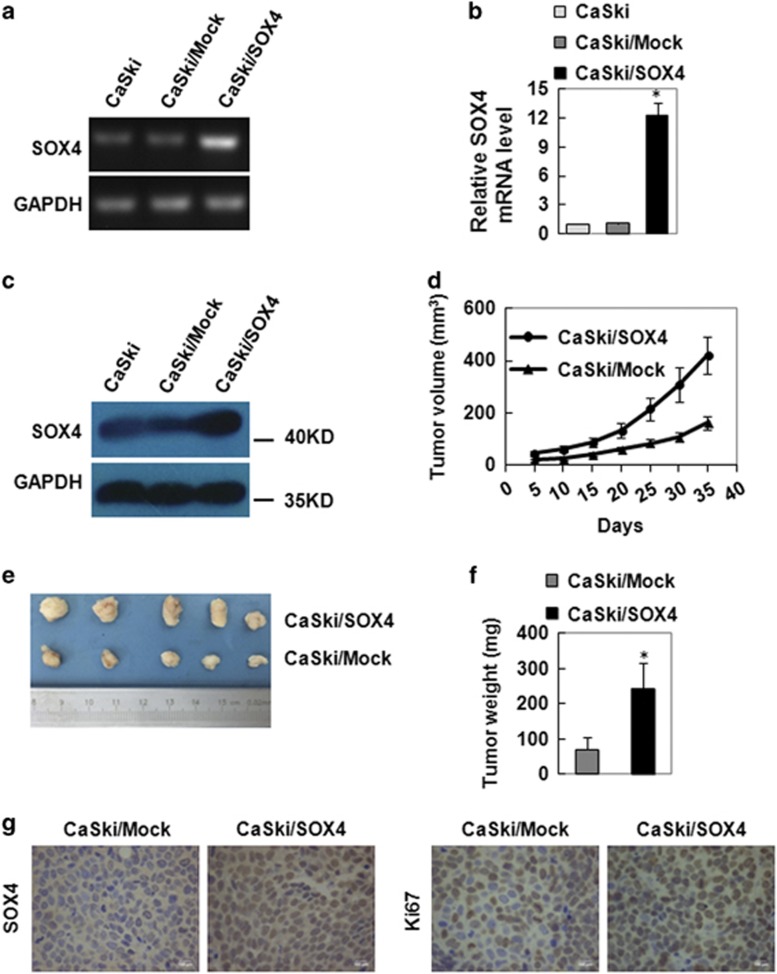
SOX4 overexpression promotes tumor growth *in vivo*. RT-PCR (**a**), qRT-PCR (**b**) and western blot (**c**) analysis of SOX4 protein in wild-type CaSki, mock-transfected CaSki (CaSki/Mock) and SOX4-overexpressed CaSki (CaSki/SOX4) cells. GAPDH served as loading controls. (**d**) *In vivo* analysis of the effect of SOX4 overexpression on CaSki tumor growth (*n*=5 in each group). (**e**) Tumor photographs from mice 35 days post implanted with CaSki/SOX4 and CaSki/Mock cells. (**f**) Weights of tumors from mice 35 days post implanted with CaSki/SOX4 and CaSki/Mock cells. (**g**) Immunohistochemistry analysis of SOX4 and Ki67 in tumors from mice 35 days post implanted with CaSki/SOX4 and CaSki/Mock cells (× 400). The data were shown as the mean±S.E.M. *P<0.05 compared with the control groups

SOX4 expression in CaSki cells was upregulated by stably transfecting a constructed SOX4-expressing plasmid. RT-PCR results showed that the mRNA level of SOX4 was upregulated by SOX4 gene transfer (Figure 2a). qRT-PCR results showed that the mRNA levels of CaSKi/SOX4 was 12.33 folds compared with that of CaSki/Mock (Figure 2b), and the upregulation was significant. Furthermore western blot results also showed that the SOX4 protein levels in CaSki/SOX4 was upregulated compared with that in wild-type CaSki, or CaSki/Mock (Figure 2c). Next, CaSki/SOX4 and CaSki/Mock cells were injected subcutaneously into nude mice, respectively. SOX4-overexpressed xenograph tumors grew faster than the tumors from mock-transfected CaSki cells (Figure 2d). At the end of experimental period, the tumor size from CaSki/SOX4 was larger than that from CaSki/Mock (Figure 2e), and the weights of tumors were 420.88±71.62 and 160.14±26.33 mg, respectively (Figure 2f; *P*<0.05). These results indicated that the SOX4 protein enhanced tumor growth of CC cells *in vivo*.

To further explore why SOX4 enhanced the tumor formation of CC cells *in vivo*, the expression of Ki67, which is a marker for proliferation, was evaluated by immunohistochemistry in the tumor tissues xenographed by the CaSki/Mock and CaSki/SOX4 cells. Ki67 staining was found to be expressed much stronger in the tumor tissues from CaSki/SOX4 cells than that in the tumor tissues from CaSki/Mock cells (Figure 2g). These results suggested that SOX4 enhanced tumor formation by promoting the proliferation of CC cells *in vivo*.

### SOX4 enhanced the growth and proliferation of cervical cancer cells *in vitro*

**Figure 3:**
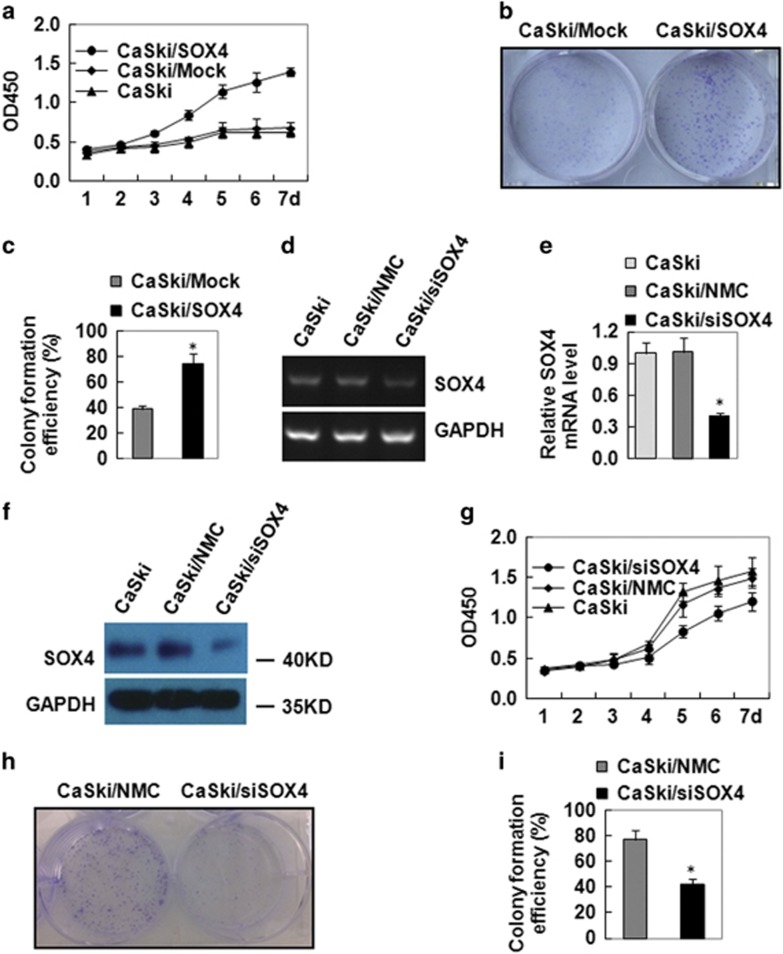
SOX4 overexpression enhances the proliferation of cervical cancer cells *in vitro.* (**a**) CCK-8 analysis of the effect of SOX4 overexpression on cell proliferation *in vitro*. (**b**) Colony formation assay analysis of the role of SOX overexpression on CaSki tumorigenicity. (**c**) Quantitative data of RT-PCR (**d**), qRT-PCR (**e**) and western blot (**f**) analysis of SOX4 expression in CaSki, CaSki/NMC and CaSki/siSOX4 cells. GAPDH served as the loading control. (**g**) CCK-8 analysis of the effect of SOX4 downexpression on cell proliferation *in vitro*. (**h**) Colony formation assay analysis of SOX downexpression on CaSki tumorigenicity. (**i**) Quantitative data of (**h**). Data were presented as the mean±S.E.M

To confirm the effect of SOX4 on the cell growth and proliferation of CC cells *in vitro*, CCK-8 assay and colony formation assay were used. CCK-8 assay results showed that CaSki/SOX4 cells grew much more quickly than CaSki/Mock cells (Figure 3a). Colony formation assay results showed that the colonies of CaSki/SOX4 cells were bigger than that of CaSki/Mock cells (Figure 3b), and the colony formation rate increased from 68.8±6.2 to 86.4±8.3% (Figure 3c).

Furthermore, we inhibited SOX4 expression by RNA interference in CaSki cells (CaSki/siSOX4). RT-PCR results showed that RNA interference downregulated the mRNA levels of SOX4 (Figure 3d). qRT-PCR data showed that the downregulation was >60% (CaSki/siSOX4) compared with the controls (CaSki and CaSki/NMC; Figure 3e). Western blot results showed that RNA interference also decreased the protein levels of SOX4 (Figure 3f). Similarly, CaSki/siSOX4 cells were found to have much slower growth rate than CaSki/NMC cells tested by CCK-8 assays (Figure 3g). Furthermore, the colony formation assay results showed that CaSki/siSOX4 cells displayed smaller colony (Figure 3h) and much lower colony formation rate (42.4±4.3%) than their control CaSki/NMC cells (76.8±7.5% Figure 3i). All these data indicated that SOX4 downregulation inhibited the growth and proliferation of CC cells *in vitro*.

### SOX4 accelerated the progress of cervical cancer cells from G0/G1 into S phase

**Figure 4:**
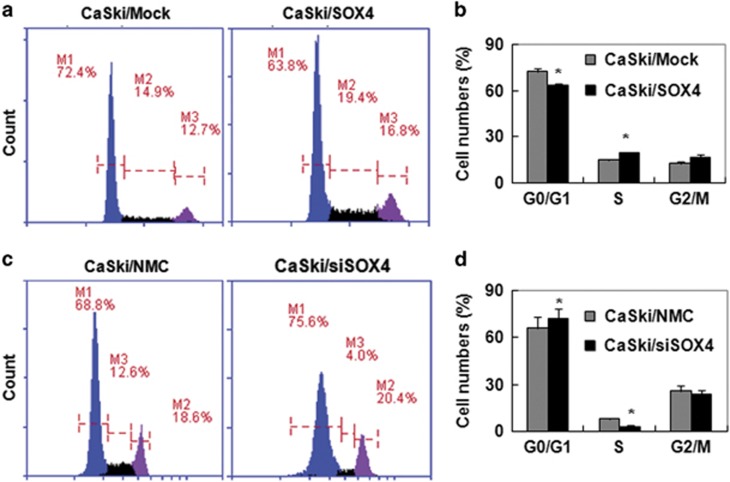
SOX4 accelerates the progress of the cell cycle from G0/G1 phase into S phase in cervical cancer cells. (**a**) PI staining analysis of the cell cycles of CaSki/Mock and CaSki/SOX4 cells. (**b**) Quantitative data of **a**. (**c**) PI staining analysis of the cell cycles of CaSki/NMC and CaSki/siSOX4 cells. (**d**) Quantitative analysis of **c**. The data were analyzed and are presented as the mean±S.E.M. *P<0.05

To investigate how SOX4 protein affects cell proliferation of CC cells, fluorescence-activated cells sorting (FACS) was used to analyze the cell cycle of SOX4-modified CaSki cells. The results showed that the population of CaSki/SOX4 cells in G0/G1 phase and S phase were 63.8% and 19.4%, respectively, whereas the population of CaSki/Mock cells in G0/G1 phase and S phase were 72.4% and 14.9%, respectively (Figures 4a and b), suggesting that SOX4 expression promoted the cell cycle progress of CC cells from the G0/G1 phase into the S phase. Furthermore, Figure 4c and d showed that 74.6% of the CaSki/siSOX4 cells were in the G0/G1 phase, and 5.1% were in the S phase cells, whereas of the CaSki/NMC cells, 67.9% were in the G0/G1 phase and 11.7% were in the S phase suggesting that a lack of SOX4 might inhibit the cell cycle progress of CC cells from the G0/G1 phase into the S phase. All of these results indicated that SOX4 was a positive regulator of the cell cycle in CC cells.

### SOX4 increased the resistance of cervical cancer cells to cisplatin-induced cell death

**Figure 5:**
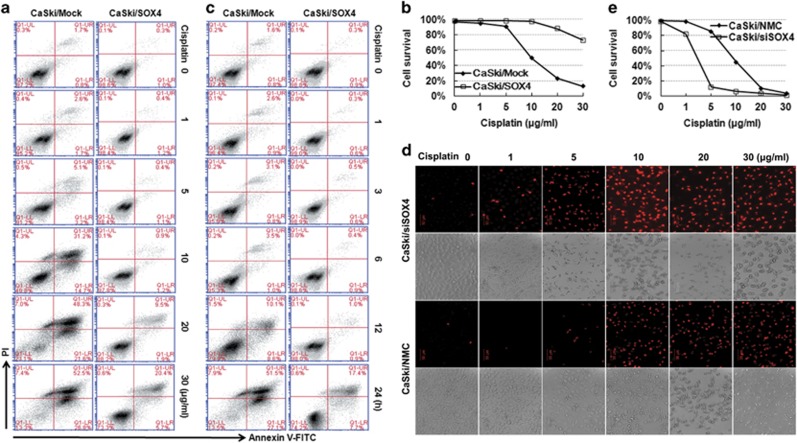
The sensitivity of SOX4-modified CaSki cells to cell death induced by cisplatin. (**a**) Annexin V-FITC and PI staining analysis of the effect of SOX4 overexpression on cell death induced by 0–30 *μ*g/ml cisplatin for 24 h. (**b**) Quantitative data of **a**. (**c**) Annexin V-FITC and PI staining analysis of the effect of SOX4 overexpression on cell death induced by 30 *μ*g/ml cisplatin for 0–24 h. (**d**) PI staining analysis of the effect of SOX4 downregulation on cell death induced by 0–30 *μ*g/ml cisplatin for 24 h. (**e**) Quantitative data of **b**. The data were analyzed and were presented as the mean±S.E.M. Scale bar, 50 *μ*m

The resistance to cisplatin, a class of platinum-containing anti-cancer drugs that ultimately triggers apoptosis, was measured in SOX4-modified CaSki cells. The FACS showed that the apoptosis of CaSki/SOX4 cells reduced significantly compared with that of CaSki/Mock cells induced by the same concentrations of cisplatin (10, 20 and 30 *μ*g/ml) for 24 h (Figure 5a), and the median lethal dose of cisplatin were >30 *μ*g/ml, or ~10 *μ*g/ml in CaSki/SOX4 cells, or CaSki/Mock cells, respectively (Figure 5b). Then we detected the time course of cisplatin-induced apoptosis in CaSki cell. When CaSKi/Mock and CaSki/SOX4 cells were treated with 30 *μ*g/ml cisplatin for serious time periods, the results showed that the apoptosis of CaSki/SOX4 cells reduced obviously compared with that of CaSki/Mock cells after 12 h (Figure 5c).

In addition, we detected the resistance to cisplatin in SOX4 knockdown CaSki cells. The dead cells of CaSki/siSOX4 cells and CaSki/NMC cells induced by cisplatin can not be measured by Annexin V- FITC/PI staining because green fluorescent protein was highly expressed in these cells. So PI alone staining was used to distinct the live and dead cells. PI staining results showed that the dead cells (PI positive) of CaSki/siSOX4 cells rose markedly compared with that of control CaSki/NMC cells (Figure 5d), and the median lethal dose of cisplatin were about 3 *μ*g/ml, or 10 *μ*g/ml in CaSki/siSOX4 cells, or CaSki/NMC cells, respectively (Figure 5e). All of these results indicated that SOX4 had a chemotherapy drug resistance role in CC cells.

### SOX4 increased the expression of the multidrug-resistant gene ABCG2 *in vitro* and *vivo*

**Figure 6:**
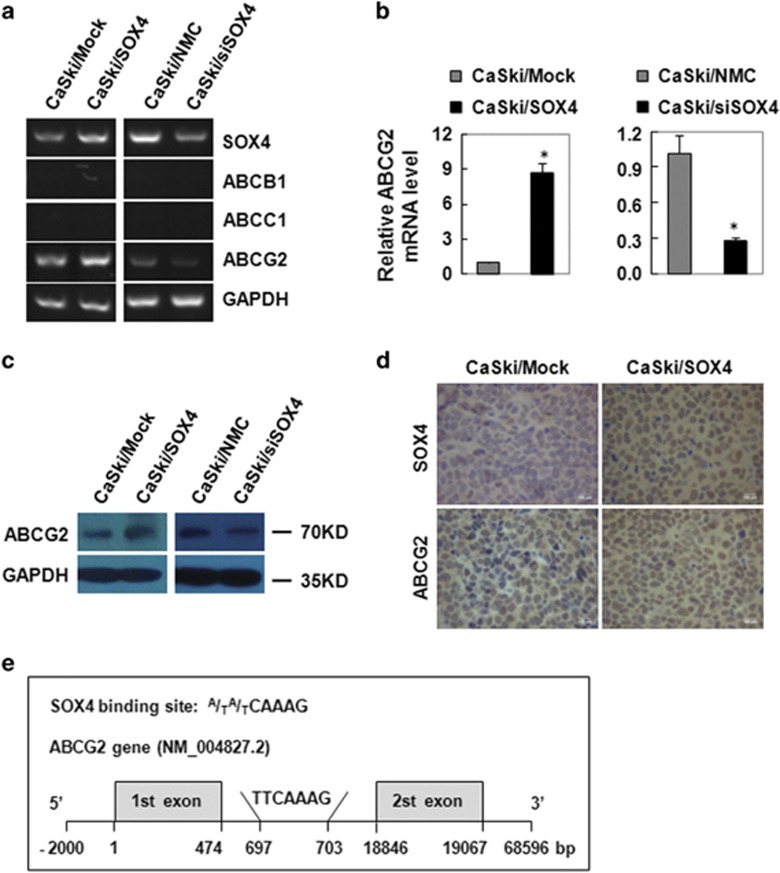
SOX4 regulated the expression of ABCG2 in SOX4-modified CaSki cells. RT-PCR (**a**), qRT-PCR (**b**) and western blot (**c**) analysis of the effect of SOX on ABCG2 mRNA and protein expression. (**d**) Immunohistochemistry analysis of SOX4 and ABCG2 expression in tumor xenografts from the mice implanted with CaSki/Mock and CaSki/SOX4 cells (× 400). (**e**) The diagram of SOX4-binding site with ABCG2. In the gene diagram, the ABCG2 gene (horizontal line) and base position (vertical lines) are drawn to scale. Data were shown as the mean±S.E.M. *P<0.05

To explore the molecular mechanism involved in the function of SOX4 on the resistance to cisplatin of CC cells, the mRNA levels and protein levels of multidrug resistant gene (ABCB1, ABCC1 and ABCG2) were examined by RT-PCR, qRT-PCR and western blot in SOX4-modified CC cells. RT-PCR results showed that the mRNA levels of ABCG2 elevated obviously in CaSki/SOX4 cells compared with that of control CaSki/Mock cells, and reduced significantly in CaSki/siSOX4 cells compared with that in control CaSki/NMC cells, whereas the mRNA levels of ABCB1 and ABCC1 were not detected in SOX4-modified CaSki cells (Figure 6a). qRT-PCR results showed that the mRNA levels of ABCG2 increased to 8.75 folds in SOX4 overexpression CaSki cells and reduced to 0.28 folds in SOX4 knockdown CaSki cells compared with that of their own control cells (Figure 6b). The protein levels of ABCG2 were consistently regulated with their mRNA levels in SOX4-modified CaSki cells (Figure 6c). All these results confirmed that SOX4 increased the expression of the multidrug resistant gene ABCG2 at both mRNA and protein levels in CC cells.

To further determine the relationship between SOX4 and ABCG2 *in vivo*, the expression of SOX4 and ABCG2 was evaluated by immunohistochemistry in the tumor tissues xenographed by the CaSki/Mock and CaSki/SOX4 cells. The results showed that SOX4 expression was upregulated in CaSki/SOX4 cells compared with that in CaSki/Mock cells (Figure 6d). Consistently, ABCG2-positive staining localized in membrane and/or cytoplasm and was found to be much stronger in the tumor tissues formed by the SOX4-overexpressing CaSki cells than that in the tumors formed by the control cells (Figure 6d). These data suggested that the expression level of ABCG2 was upregulated with the upregulation of SOX4. In addition, we blast the SOX4-binding site (^A^/_T_^A^/_T_CAAAG) on the ABCG2 gene sequence. In the region of 2000 bp upstream and 500 bp downstream of the first exon in ABCG2 gene, we found one binding site in the 693 bp and 703 bp (TTCAAAG), which was in the first intron (Figure 6e). These data demonstrated that SOX4 may regulate ABCG2 transcription by binding to the site.

## Discussion

Aberrant expression of SOX4 has been identified in several human cancers, and it is often associated with the progression of cancer. In non-small cell lung cancer,^27^ breast cancer,^28^ cholangiocarcinoma^29^ and prostate cancer patients,^30^ increased SOX4 expression is a biomarker for malignant status and poor prognosis. SOX4 is highly expressed in human acute lymphoblastic leukemia cells and SOX4/Tcf7l1 is a functional axis that promotes the progression of BCR-ABL+ acute lymphoblastic leukemia.^31^ In our study, we found that the expression of SOX4 was upregulated in three CC cell lines, particularly in CaSki cells, and in cervical squamous carcinoma tissues, suggesting that SOX4 may be involved in the progression of CC.

To further investigate the role of SOX4 in the progression of CC, we overexpressed SOX4 in CaSki cells and found that SOX4 overexpression in xenograph tumors grew faster than that of the control group. Also, Ki67 staining was significantly stronger in the tumor tissues formed by the SOX4-overexpressing CaSki cells than that formed by the control CaSki cells, suggesting that SOX4 enhanced tumor formation by promoting the proliferation of CC cells *in vivo*. *In vitro*, the overexpressed SOX4 promoted, whilst the downregulated SOX4 inhibited, the growth and proliferation of CaSki cells. SOX4 roles for promoting tumor progression by accelerating cell growth and proliferation have been reported previously. Zhou *et al.*^32^ demonstrated that overexpressed SOX4 promotes cell proliferation and invasion in SGC-7901/MKN45 gastric cancer cells. Zhou *et al.*^33^ suggested that downregulated SOX4 expression suppresses cell proliferation, metastasis and induces apoptosis in Xuanwei female lung cancer patients. Koumangoye *et al.*^34^ documented that SOX4 promotes esophageal tumor cell proliferation and invasion by silencing miR-31 via activation and stabilization of a corepressor complex with EZH2 and HDAC3.

We then investigated how SOX4 affects cell proliferation of CC cells. FACS analysis suggested that SOX4 promoted the cell cycle progress of CC cells from G0/G1 phase to S phase, suggesting that it is a positive regulator of the cell cycle progress in CC cell. Similarly, SOX4 was reported to be required for the KLF5-mediated cell proliferation, and the expression of cell cycle regulators in lung cancer.^35^ Although we do not investigate the potential mechanisms for these effects of SOX4 on CC tumorigenesis, some pathways have been described, for example, SOX4 directly bound to cAMP response element-binding protein (CREB) and cooperated with CREB to enhance myeloid cell proliferation.^36^ However, another group reported that SOX4 inhibits primary glioblastoma multiforme (GBM) cell growth and induces G0/G1 cell cycle arrest through Akt-p53 axis, and high SOX4 expression was significantly associated with good prognosis of GBM.^37^ So, the function of SOX4 is pleiotropic and is various in different cancer types.

Cisplatin is one of the primary chemotherapeutic drugs in cancer treatment. Chemoresistance of cancer is often mediated by ABC transporters.^15^ Of the 48 human ABC transporters, ABCB1, ABCC1 and ABCG2 are most often associated with multidrug resistance.^15^ Other ABC transporters have been implicated in drug resistance, but these transporters have highly specialized roles in normal physiology and are less likely to have a role in drug resistance in cancer cells. Chemotherapeutic drug resistance is a main cause of chemotherapy failure. The resistance to cisplatin has been reported to be related to the upregulated expression of ABCG2 in various cancers, such as breast cancer,^38^ esophageal carcinoma cells^39^ and lung cancer cells.^40^ In the present study, we found that with proper concentration cisplatin induced the death of CaSki cells, and SOX4-modified CaSki cells exerted different death rates when treated by cisplatin. In CaSki cells with SOX4 overexpression, the cell death induced by cisplatin reduced sharply. However, when SOX4 was downregulated, the cell death increased obviously. These results demonstrated that transcript factor SOX4 regulated the sensitivity of CC cells to cisplatin, and SOX4 may be a target for sensitization in chemotherapy of CC.

The regulation of ABCG2 was influenced by a series of factors. It has been reported that progesterone^38^ and aryl hydrocarbon receptor^39^ upregulate ABCG2 expression, whilst DNA methylation represses ABCG2 expression in human renal carcinoma,^41^ and in multiple myleoma cell lines.^42^ Cytokines and growth factors have also been reported to alter the expression of the ABCG2. Transforming growth factor-beta decreases ABCG2 gene expression in MCF-7 cells.^43^ Tumor necrosis factor-alpha or interleukin 1 beta decreases mRNA and protein expression of ABCG2, whereas insulin-like growth factor II increases ABCG2 expression in primary term trophoblasts.^44^ It has been reported that the number of ABCG2-positive cells reduce in Akt1-deficient mice and when ABCG2-positive cells from normal mice were incubated with the phosphotidylinositol 3-kinase inhibitor LY294002, ABCG2 protein was translocated from the plasma membrane to the cytoplasm.^45^ In the present study, we found that the gene and protein levels of ABCG2 were regulated by SOX4 expression. An increased expression of ABCG2 was found in SOX4-overexpressed CaSki cells, whereas a reduced expression of ABCG2 was found in SOX4 knockdown CaSki cells. In mouse xenograft tumors, immunohistochemistry analysis denoted that the expression of ABCG2 was consistent with the SOX4 expression. Furthermore, the SOX4-binding site was found in ABCG2 gene. All these data demonstrated that SOX4 induces resistance to cisplatin in CaSki cells by regulating ABCG2. The transcription factor SOX4 may directly binds to ABCG2 gene to regulate the ABCG2 transcript, which remains to be confirmed in further study.

In terms of our study, SOX4 is an unfavorable biomarker of malignant status and poor prognosis in CC. So it may bring some enlightenments and help us in clinical practice process. For example, we can detect the expression levels of SOX4 in CC tissues before we determine the therapy strategy for CC patients with chemotherapeutic drug cisplatin. If abnormally increased SOX4 was found, it hinted that the resistance to cisplatin may exist and another therapeutic schedule should be considered. Of course, combined detection of the protein levels of SOX4 and ABCG2 will be more reliable. Recently reported that microRNAs, such as miR-129-5p,^46^ miR-138,^47^ miR-129^48^ and miR-335,^49^ can directly target the 3′UTR of SOX4 genes and repress the post-transcriptional activities of SOX4. So, for the CC patients who resist to cisplatin because of abnormally elevated SOX4, it will be a good way to elevate the microRNA levels to regulate SOX4 expression, and the decreased SOX4 expression will abolish the resistance to cisplatin.

In conclusion, we found that SOX4 was upregulated in cervical cancer cells. The regulation of SOX4 affected the tumor proliferation via modulation of cell cycle. SOX4 regulation also affected drug sensitivity via modulation of multiple drug resistant gene AGCG2, suggesting that SOX4 may be an indicator for clinical drug selection and CC chemotherapy.

## Materials and Methods

### Cell culture

Human normal cervical epithelial cells H8 and human cervical cancer cells CaSki, HeLa and SiHa were maintained by our own lab and cultured in Dulbecco's modified Eagle's medium (DMEM) (Gibco, Invitrogen, VIC, Mount Waverley, Australia) supplemented with 10% bovine calf serum (Gibco, Invitrogen, VIC, Mount Waverley, Australia) and antibiotics. All cells were cultured in a humidified atmosphere with 5% CO_2_ at 37 °C.

### RT-PCR

Total RNA was extracted from 1–2 × 10^6^ cells using Trizol (Life Technologies, Gaithersburg, MD, USA) according to the manufacturer's instructions. mRNA was reverse transcribed with RevertAid (MBI Fermemtas, Burlington, Ontario, Canada) at 42 °C for 60 min, and the resulting cDNA was subjected to PCR (94 °C for 1 min followed by 20–30 cycles at 94 °C for 30 s, 60 °C for 30 s, 68 °C for 90 s and an extension cycle for 10 min at 68 °C). PCR products were separated on 1.0% agarose gels and visualized with GelRed. The primer pairs are listed (5′–3′) as follows:

GAPDH-F: AATCCCATCACCATCTTCCA

GAPDH-R: CCTGCTTCACCACCTTCTTG

ABCB1- F: CCGAACACATTGGAAGGAAA

ABCB1-R: CCATAGGCAATGTTCTCAGCA

ABCC1-F: TGGACTAACGGCTAACCTGGA

ABCC1-R: TAAGCAACCAACACTGCTTTG

ABCG2-F: TCTCTTCTTCCTGACGACCAA

ABCG2-R: AAACCACACTCTGACCTGCTG

### Quantitative real time RT-PCR (qRT-PCR)

The qRT-PCR was performed as described by Sun *et al.*^50^ Briefly, total RNA was isolated and reversely transcribed as above. The cDNA was amplified using TaqMan Universal PCR master mix (Applied Biosystems, Foster City, CA, USA) and an ABI Prism 7500 sequence detection system (Applied Biosystems). Amplification of the target genes was normalized using the amplification levels of GAPDH as the endogenous control. The efficiency of the PCR was tested by amplification of the target from serially diluted cDNA generated from the reverse transcription of a stock set of human RNA. Data analysis and calculations were performed using the 2^−ΔΔCT^ comparative method, as described by the manufacturer.

### Western blot

Rabbit anti-human antibodies against SOX4 (Abcam, Cambs, England), ABCG2 (Santa Cruz, Santa Cruz, CA, USA) and GAPDH (Beyotime, Shanghai, China) were used in western Blot. A total of 1–2 × 10^6^ cells were lysed in 200 μl RIPA lysis buffer containing a protease inhibitor cocktail tablet (Roche, Basel, Switzerland). The cell lysate was centrifuged at 12 000 × *g* at 4 °C for 5 min. Equivalent amounts of protein were electrophoresed on 10% SDS-PAGE gels and transferred onto Immobilon P membranes (Millipore, Bedford, MA, USA). The membranes were blocked by incubating with 3% nonfat dry milk for 90 min at 37 °C and then incubated with primary antibodies (1 : 200–1000) in PBST (containing 0.01% Tween 20) overnight at 4 °C. After incubation with a horseradish peroxidase-conjugated secondary antibody (1 : 2000), the protein bands were detected with SuperSigna Chemiluminescent Substrate Stable Peroxide Solution (Pierce, Rockford, IL, USA) and BIOMAX-MR film (Eastman Kodak, Rochester, NY, USA).

### Cell transfection

The SOX4 expression plasmid pENTER/SOX4, containing SOX4 open reading frame, and the negative control plasmid pENTER were purchased from Vigene Biosciences (Shandong, China). The SOX4 interference plasmid Lv-shRNA-GP/SOX4, containing the target sequence (5′-AGCGACAAGATCCCTTTCATT-3′) against SOX4 gene, and the negative mammalian control plasmid Lv-shRNA-GP/NMC containing the sequence (5′-CAACAAGATGAAGAGCACCAA-3′) that did not target any known genes were purchased from Longqian Biotech (Shanghai, China). The desired sequence was confirmed by direct DNA sequencing.

CaSki cells grown to 70–80% confluence were transfected with pENTER (CaSki/Mock), pENTER/SOX4 (CaSki/SOX4), Lv-shRNA-GP/NMC (CaSki/NMC) or Lv-shRNA-GP/SOX4 (CaSki/siSOX4) using Lipofectamin 2000 (Life Technologies), and harvested 48 h after the transfection, followed by limited dilution in 96-well plates for the generation of single cell clones. Three weeks later, the mRNA levels of SOX4 in the cell clones were measured by RT-PCR and qRT-PCR analysis. The protein levels of SOX4 were measured by western blot.

### Xenograft mouse experiment

A total of 1 × 10^7^ cells in 200 *μ*l PBS were injected subcutaneously into 6-week-old female BALB/c nude mice (SLAC Laboratory Animal Company, Changsha, China). Five mice per group were used in each experiment. Tumor dimensions were measured every 5 days, and the volumes were calculated by the standard formula: length × width^2^/2.^51^ After 45 days, the mice were killed by cervical vertebra dislocation, and the weights of the tumors were measured after being dissected out.

### Immunohistochemistry

Human cervical tissue microarray was purchased from Shanghai Outdo Biotech Company (Shanghai, China). Mouse xenograft tumor tissues were fixed in neutral buffered 5% formalin, paraffin embedded, and 4-*μ*m sections were collected. All sections of tissue samples were deparaffinized and rehydrated and then treated with pH 6.0 citrate antigen repair buffer (Human cervical tissue microarray) or pH 8.0 EDTA antigen repair buffer (Mouse xenograft tumor tissues) in a microwave oven with middle temperature for 8 min, and let stand for 8 min followed by low temperature for 7 min. Sections were incubated with 3% H_2_O_2_ at room temperature away from light for 25 min and then blocked with 10% rabbit serum for 30 min. After washing three times with PBS at room temperature, the sections were incubated with the following primary antibodies at 4 °C overnight: anti-SOX4 (1 : 100, Abcam), anti-ABCG2 (1 : 100, Santa Cruz) and anti-Ki67 (1 : 200, Wuhan Goodbio Technology Company, Wuhan, China). After washing three times with PBS, the sections were then incubated with a horseradish peroxidase-conjugated secondary antibody at room temperature for 50 min and visualized with the treatment of 3,3′-diaminobenzidine under the microscope (Leica, Solms, Germany). The nuclei were counterstained with hematoxylin. Negative controls were performed in the same manner, except that PBS was used as a substitute for the primary antibody. The IHC was examined by two separate researchers using microscope in five randomly selected representative fields at × 40 magnification. The evaluation of SOX4 staining were performed using the immunoreactivity scores. The score was determined by multiplying the staining intensity by the staining extent. The staining intensity for SOX4 was scored as 0 (negative), 1 (weak), 2 (moderate) and 3 (strong). The staining extent was scored as 0 (0%), 1 (1–25%), 2 (26–50%), 3 (51–75%) and 4 (76–100%) according to the percentage of positively stained cells.^52^ The SOX4 staining was defined into two categories according to immunoreactivity scores: negative (1–4) and positive (5–12).

### Colony formation assay

The effect of SOX4 on the colony formation of CaSki cells was analyzed by colony formation assay. Briefly, cells (500 cells per well) in 6-well plates were cultured in 10% FBS DMEM at 37 °C 5% CO_2_ for 2 weeks. The cell colonies were washed twice with PBS, and then fixed with 4% paraformaldehyde for 20 min and stained with 0.1% gentian violet solution for 20 min. Individual clones with >50 cells were counted. Clone forming efficiency for individual type of cells was calculated according to the following formula: clone forming efficiency=number of colonies/number of inoculated cells × 100%.

### Cell proliferation assays

Cell proliferation was assessed by the CCK-8 assay. Briefly, 2000 cells were seeded to each well of 96-well plates in triplicate. After incubation for indicated time, 5 *μ*l CCK-8 (DOJINDO, Japan) was added to each well and incubated for 1 h at 37 °C. Cell viability was determined by measuring the absorbance at 450 nm. The experiments were performed at least three times.

### Flow cytometry analysis of cell cycle

Cells were harvested and fixed with 70% cold ethanol at 4 °C overnight. After being washed in PBS, the cells were incubated in 1 ml of staining solution (0.5 *μ*g/ml PI; 100 *μ*g/ml RNaseA) at room temperature for 30 min. Then, the samples were measured by FACS (BD Biosciences, Franklin Lakes, NJ, USA).

### Flow cytometry analysis of cell apoptosis

The apoptosis of CaSki/SOX4 cells and CaSki/Mock cells were detected by Annexin V- FITC/PI staining. In general, 1–2 × 10^6^ cells were washed twice with PBS and then labeled with Annexin V- FITC and PI in binding buffer (JiaMei, Beijing, China), according to instructions provided by the manufacturer. Fluorescence signals of FITC and PI were detected on a FACS (BD Biosciences). The log of Annexin V- FITC fluorescence was displayed on the *x*-axis, and the log of PI fluorescence was displayed on the *y*-axis.

### PI staining of dead cell

The death of CaSki/siSOX4 cells and CaSki/NMC cells were detected by PI staining. In general, cells were cultured overnight and added to 0–30 *μ*g/ml cisplation (Tocris, Bristol, UK) for 24 h. The culture supernatant was discarded and the cells were incubated in 1 ml PBS containing 0.5 *μ*g/ml PI (JiaMei) and 100 *μ*g/ml RNaseA at room temperature for 30 min. Then, the white light and fluorescence images were captured by fluorescence microscope (Leica) at the same time.

### Statistical analysis

All experiments were performed at least three times, and results were expressed as the mean±S.E.M. Differences between groups were examined using Student's *t*-test, and *P*-values <0.05 were considered to be significant (*n*=3 in each qRT-PCR test).

## Abbreviations

ABC: ATP-binding cassette
CC: cervical cancer
CREB: cAMP response element-binding protein
DMEM: Dulbecco's modified Eagle's medium
GBM: glioblastoma multiforme
SOX: sex-determining region Y (SRY)-related high-mobility-group (HMB) box transcription factor
